# Predictors of chronic food insecurity among adolescents in Southwest Ethiopia: a longitudinal study

**DOI:** 10.1186/1471-2458-12-604

**Published:** 2012-08-03

**Authors:** Tefera Belachew, David Lindstrom, Abebe Gebremariam, Challi Jira, Megan Klein Hattori, Carl Lachat, Lieven Huybregts, Patrick Kolsteren

**Affiliations:** 1 Department of Population and Family Health, College of Public Health and Medical Sciences, Jimma University, PO.Box:1104, Jimma, Ethiopia; 2 Department of Food Safety and Food Quality, Faculty of Bioscience Engineering, Ghent University, Coupure Links 653, B- 9000 Ghent, Belgium; 3 Department of Sociology, Brown University, Box 1916, Providence, RI, 02912, USA; 4 Department of Health Planning and Health Services Management, College of Public Health and Medical Sciences, Jimma University, PO.Box:378, Jimma, Ethiopia; 5 Nutrition and Child Health Unit, Department of Public Health, Institute of Tropical Medicine, Nationalestraat 155, 2000 Antwerpen, Belgium

**Keywords:** Chronic food insecurity, Adolescent, Buffering, Ethiopia, Longitudinal

## Abstract

**Background:**

Evidence on the differential impacts of the global food crisis as it translates into chronic food insecurity locally is essential to design food security interventions targeting the most vulnerable population groups. There are no studies on the extent of chronic food insecurity or its predictors among adolescents in developing countries. In the context of increased food prices in Ethiopia, we hypothesized that adolescents in low income urban households are more likely to suffer from chronic food insecurity than those in the rural areas who may have direct access to agricultural products.

**Methods:**

This report is based on data from the first two rounds of the Jimma Longitudinal Family Survey of Youth (JLFSY). Both adolescents and households were selected using a stratified random sampling method. A total of 1911 adolescents aged 13-17 years were interviewed on their personal experiences of food insecurity both at baseline and at year two. Multivariable logistic regression analyses were used to compare chronic adolescent food insecurity by household income, household food insecurity, and socio-demographic variables after one year of follow-up.

**Results:**

Overall, 20.5% of adolescents were food insecure in the first round survey, while the proportion of adolescents with food insecurity increased to 48.4% one year later. During the one year follow up period, more than half (54.8%) of the youth encountered transient food insecurity – that is, either during the first or the second round survey. During the follow up period, 14.0% of adolescents had chronic food insecurity (i.e. were food insecure at both rounds). Multivariable logistic regression analysis showed that adolescents in the urban households with low (OR = 1.69, P = 0.008) and middle (OR = 1.80, P = 0.003) income tertiles were nearly twice as likely to suffer from chronic food insecurity compared with those in high income tertile, while this was not the case in rural and semi-urban households. Female sex of adolescents (P < 0.01), high dependency ratio (P < 0.05) and household food insecurity (P < 0.001) were independent predictors of chronic adolescent food insecurity in urban, semi-urban, and rural areas, while educational status of the adolescents was negatively associated with chronic food insecurity (OR = 0.047, P = 0.002) in urban areas.

**Conclusions:**

In the context of increased food prices, household income is an independent predictor of chronic food insecurity only among adolescents in the low income, urban households. Female gender, educational status of primary or less and being a member of households with high dependency ratio were independent predictors of chronic food insecurity in urban, semi-urban, and rural areas. The fact that the prevalence of chronic food insecurity increased among adolescents who are members of chronically food insecure urban households as income tertiles decreased suggests that the resilience of buffering is eroded when purchasing power diminishes and food resources are dwindling. Food security interventions should target urban low income households to reduce the level of chronic food insecurity and its consequences.

## Background

As a consequence of the recent rise in food prices [1-3], the proportion of people experiencing food insecurity has increased dramatically [3]. Although food prices have fallen since their peak in mid-2008, they still remain high by historic standards [4]. The food price increases in Ethiopia were larger than the increase in world markets and those observed in most other African countries [5,6]. In Ethiopia, from 2007 to 2008 there was a 100% increase in wheat and teff prices; and maize and meat prices increased by 180% and 50%, respectively [1]. The increase in food prices has led to a reduction in the quality and the quantity of food consumed, particularly amongst vulnerable households who spend a large share of their income on food [1,7,8]. Protracted lack of adequate access to food among these vulnerable groups induces chronic food insecurity [9]. Unlike transient food insecurity, which is temporary shortage of food, chronic food insecurity is a long-term challenge, associated with poverty and lower socioeconomic status [6,10,11].

Chronically food insecure individuals are exposed to long-term inadequate diets caused by the inability to acquire food [12]. When food prices increase, households that do not produce food themselves [e.g. many urban households] must acquire food in other ways, largely by purchasing food. As the ability to purchase food depends on household income, the rise in the food price can negatively impact on the purchasing power of households [1,6,12-14]. Therefore, strategies to cope with food insecurity include reduction of meal frequency and quantity, resorting to less diversified and lower quality cheaper foods [15,16]. As a result of this prolonged limited access to nutrients, chronic food insecurity could have more deleterious consequences compared to transient food shocks. Moreover the fact that adolescents are in a period of rapid growth and development with an increased demand for energy and other nutrients, and that they do not have decision making power on their access to food within the household makes them more vulnerable to the consequences of chronic food insecurity.

Since Ethiopia’s growth and development strategy identifies food security as a key issue for sustained economic growth and poverty reduction, achieving food security is high on the agenda of the Ethiopian government [17]. Thus, evidence on the differential effects of the global food crisis as it locally translates into chronic food insecurity is essential to design interventions that target the most vulnerable groups. Transient food insecurity is a common phenomenon in Ethiopia [7,8,16,18]. Previous research on adolescents in southwest Ethiopia [16,18] reported the prevalence of transient food insecurity and how adult household members buffered adolescents from its consequences [16]. However, the extent of chronic food insecurity and how adults protect adolescents against food insecurity when it becomes chronic and household income declines has not been investigated. We hypothesized that adolescents who are members of urban low income households are more likely to suffer from chronic food insecurity. This study intended to answer questions: Does the relation between household income and chronic food insecurity vary by place of residence? How resilient is intra-household buffering of adolescents from food insecurity when purchasing power is dwindling and food insecurity becomes chronic? Does this relationship vary by place of residence?

## Methods

### Study sample

This study made use of data from 1911 adolescents enrolled in the first two consecutive years of the five year longitudinal study of adolescents in Jimma zone in Southwest Ethiopia. Adolescents were included in the study from urban (Jimma city), semi-urban (small towns) and six rural communities (“Kebeles”) adjacent to the small towns. Details of the methods are presented elsewhere [19]. In brief, from 3700 households that were randomly selected, one male or female adolescent of 13-17 years old was selected from each household using a Kish table [20] with a targeted sample size of 2100 youths (700 each from urban, semi-urban and rural areas). These age groups were selected in order to capture life events as adolescents progressed to adulthood over the course of the longitudinal study. The questionnaire was pilot tested before each round of the survey with 200 adolescents in Jimma city who were not included in the main study, and modified accordingly. Interviews were conducted by full-time employees of the study who received an intensive one-week training prior to the pre-test and an additional training for one week before beginning of the actual interviews, followed by periodic training sessions during the course of the study. Supervisors followed the field procedures closely and checked the completed questionnaires every day to ensure accuracy of the data. The research team made weekly supervisory visits to the whole field team.

Ethical approval was obtained from Ethical Review Boards of both Brown University (USA) and Jimma University (Ethiopia). Both heads of households and adolescents gave informed verbal consent before interviews in both rounds of the survey.

### Measurements

The first round data were collected from mid 2005 to 2006, while the second round were finalized during the same period between 2006 to 2007. Both household and adolescent questionnaires were interviewer-administered. The household questionnaire gathered information on monthly income, residence, household food security, dependency ratio and gender of the household head. The head of the household responded to the household questionnaire.

The adolescent questionnaire gathered information on age, sex, educational status and adolescent food security. The questionnaires were translated prior to the interviews and their consistency was checked by a person who spoke both languages. Interviews were conducted in either of the two local languages (Amharic or Oromifa).

Household food insecurity was measured using a six item scale adapted from scales validated for use in developing countries [21-23]. Adolescent food insecurity was measured using a four-item scale adapted by selecting four items from the six-item household food security scale that apply to adolescents’ personal experiences as described elsewhere [24]. The two food security items that were dropped were the ones asking about other children in the household, and adolescents were not therefore expected to answer them. Adolescents were instructed to think of their own experiences, not those of the household, in answering the questions. To avoid bias in the responses related to food security, adolescents and household heads were interviewed separately. For each round of the survey, respondents who answered “yes” to any of these questions were coded as experiencing food insecurity in that survey round. Chronic adolescent food insecurity was defined here as the proportion of adolescents who responded affirmatively to at least one of the four food security item in both rounds of the survey, one year apart. Similarly, chronic household food insecurity is the proportion of households who responded affirmatively to at least one of the six food insecurity items in both rounds of the survey [9].

We used background characteristics from the first round of the survey including household income, dependency ratio, age, highest school grade completed by the adolescent, maternal education, paternal education and sex of the household head as predictor variables. Specifically, we expected that adolescents that were part of households with higher income, with lower proportions of dependent people, with higher education level or those who are male would have better access to food and be less likely to have chronic food insecurity. Household income was divided into tertiles coded as “high”, “middle” and “low”.

Household dependency ratio was calculated based on age classifications as the ratio of people who are potentially expected to be nonproductive (age groups greater than 64 and less than 15 years) to people who are expected to be potentially productive (age 15-64 years). After calculating dependency ratio for each household, it was rank ordered and divided into tertiles of “high”, “middle”, or “low” dependency ratio.

Buffering was defined as protection of adolescents from chronic food insecurity by adult household members within chronically food insecure households. In this case, buffering is the proportion food secure adolescents within chronically food insecure households.

### Data analysis

The data were entered in double, checked for missing values and outliers, and analyzed using SPSS (SPSS Inc. version 16.1, Chicago, Illinois). First, bivariate analyses were carried out to identify candidate variables for the multivariable model. Means and proportions were compared using t-test and Pearson’s Chi-square tests after checking all model assumptions. Second, to identify the predictors of chronic food insecurity, only variables that were significantly associated with chronic food insecurity in the bivariate models were entered in the multivariable logistic model. At this step, interaction between different variables was checked.

Third, since we expected income to have a different relationship between food security in the urban cash-based economy and in rural agricultural areas with more direct access to food items, and as there was an interaction between place of residence and household income (P_interaction_ = 0.01), we stratified the analysis by place of residence and fitted the model for rural, semi urban and urban areas separately.

For all scenarios, chronic adolescent food insecurity was used as the dependent variable. Covariates included sex, household income tertile, sex of head of the household, dependency ratio, sex of the head of the household and the highest school grade completed by the adolescent. Covariates were entered with a forward procedure. All tests were two-sided and P < 0.05 was considered statistically significant. We report the results as Odds Ratios (OR) and 95 percent Confidence Intervals (CI). Finally, to assess the resilience of buffering within the households, in relation to household income, we did a cross tabulation of chronic adolescent food insecurity by household income stratified by place of residence for adolescents in chronically food insecure households.

## Results

Overall, 1911 adolescents responded to both the first and the second round food security survey, while 173 were lost to follow-up. The response rate was 91.7%. There was no significant difference in food insecurity at baseline (first round) survey between adolescents who participated in the second round survey and those who dropped out.

**Figure 1:**
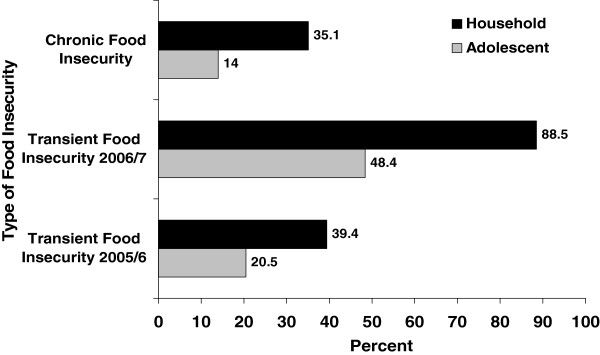
Transient and chronic food insecurity among adolescents and households in Jimma zone Southwest Ethiopia.

In the first round of the survey, 20.5% of adolescents had transient food insecurity, while the proportion of adolescents with transient food insecurity rose to 48.4% one year later. In general, more than half (54.8%) of the adolescents encountered transient food shocks either during the first or the second round survey. During the follow up period, 14.0% of adolescents were chronically food insecure, i.e., they were food insecure in both survey rounds (Figure 1).

The results of bivariate analyses showed that chronic food insecurity was associated with the background characteristics in the first round survey. The proportion of adolescents with chronic food insecurity increased as income tertiles decreased (P value for trend <0.001). Adolescents who were members of households with high dependency ratio had a higher frequency of chronic food insecurity compared to those with low dependency ratio (P = 0.002). The proportion of female adolescents (15.9%) who were chronically food insecure was significantly (P < 0.001) higher than that of males (11.1%). A higher proportion (22.6%) of adolescents who were members of food insecure households in the first round of the survey were chronically food insecure compared to 8.3% of those in food secure households (P < 0.001). Similarly, adolescents with educational status of primary level were twice as likely to be chronically food insecure compared to those with an educational status of secondary and above (14.4% vs. 7.0%, P < 0.001).

It was also observed that a higher proportion of adolescents in female headed households were chronically food insecure compared to those in male-headed households (17.5% vs. 13.2%, P = 0.038). Adolescents whose mother (P = 0.003) and father (P < 0.001) were illiterate were more often chronically food insecure than those whose parents were literate. Analysis of chronic food insecurity by seasons of the year showed that there was no significant difference (P = 0.642) in the proportion of adolescent with chronic food insecurity between the three seasons over which data were collected (Table 1).

**Table 1:** Chronic adolescent food insecurity by household food insecurity and socio-demographic characteristics at baseline

**Baseline characteristics**	**n**	**Adolescent chronic food security status**		**P**
		**Food secure**	**Food insecure**	
Household income (tertiles)				
Low	633	82.6%	17.4%	*<0.001*
Middle	653	84.1%	15.9%	
High	625	91.5%	8.5%	
Residence				
Urban	671	86.4%	13.6%	*0.383*
Semi-urban	549	84.3%	15.7%	
Rural	691	87.0%	13.0%	
Household food security status				
Food secure	1154	91.7%	8.3%	*<0.001*
Food insecure	757	77.4%	22.6%	
Sex				
Female	917	84.1%	15.9%	*0.018*
Male	994	87.8%	12.2%	
Educational status of the adolescent				
Primary (grade 0-8)	1582	85.6%	14.4%	*<0.001*
Secondary and above (grade >9)	329	93.0%	7.0%	
Dependency ratio (tertiles)				
Low	584	88.9%	11.1%	*0.002*
Middle	686	87.2%	12.8%	
High	641	82.2%	17.8%	
Household size (mean ± SD)	1911	8.4 (3.4)	8.5 (3.4)	*0.6461*
Age of adolescents in years (mean ± SD)	1911	14.7 (1.3)	14.8 (1.3)	*0.755*
Sex of the household head				
Male	1568	86.8%	13.2%	*0.038*
Female	343	82.5%	17.5%	
Maternal education				
Illiterate	1096	84.8%	15.2%	*0.003*
Primary	592	85.8%	14.2%	
Secondary and above	195	93.8%	6.2%	
Paternal education				
Illiterate	624	85.1%	14.9%	*<0.001*
Primary	773	84.5%	15.5%	
Secondary and above	420	92.4%	7.6%	
Season of the Year				
Spring	865	85.3%	14.7%	0.642
Winter	252	85.7%	14.3%	
Summer	794	86.9%	13.1%	
All	1911	86.0%	14.0%	

Jimma Longitudinal Family Survey of Youth; Round 1(2005/2006); Round 2(2006/2007).

Means and proportions were compared using t-test and Chi-square tests, respectively.

^†^Means with standard deviations in brackets are shown, unless otherwise indicated.

SD: Standard deviation.

In multivariable logistic regression model, after adjusting for the effect of the other variables, adolescents in the low (OR = 1.61, P < 0.026) and middle (OR = 1.74, P = 0.006) household income tertiles were nearly twice as likely to suffer from chronic food insecurity compared to those in households with high income tertile. Similarly, adolescents who were members of urban (OR = 1.70, P = 0.006) and semi-urban (OR = 1.70, P = 0.003) households were twice as likely to report chronic food insecurity compared to rural ones. Likewise, female adolescents (OR = 1.46, P = 0.006) and adolescents who live in food insecure households (OR = 2.86, P < 0.001) were more likely to suffer from chronic food insecurity. On the contrary, adolescents who had educational status of primary were nearly twice as likely to have chronic food insecurity (OR = 1.97, P = 006) compared to those with an educational level of secondary or above after adjusting for other covariates. Dependency ratio was significantly associated with the odds of being chronically food insecure. Adolescents who were members of households with high tertile of dependency ratio were nearly 1.6 times as likely (P = 0.011) to report chronic food insecurity compared with those in low dependency ratio (Table 2).

**Table 2:** Multivariable logistic regression predicting the odds of being chronically food insecurity among adolescents in Jimma zone Southwest Ethiopia

**Predictors**	**AOR [95% CI]**	**P**
Sex		
Male	1.00	0.006
Female	1.46 [1.12, 1.92]	
Educational status of adolescents		
Secondary and above (>grade 9)	1.00	0.006
Primary (grade 0-8)	1.97 [1.22, 3.18]	
Household income (tertiles)		
Low	1.61 [1.06, 2.43]	0.026
Middle	1.74 [1.18, 2.58]	0.006
High	1.00	
Dependency ratio (tertiles)		
Low	1.00	0.535
Middle	1.12 [0.78,1.61]	0.011
High	1.61 [1.12, 2.31]	
Sex of the household head		
Male	1.00	0.608
Female	1.10 [0.77, 1.58]	
Place of residence		
Urban	1.70 [1.17, 2.48]	*0.006*
Semi-urban	1.70 [1.20, -2.42]	*0.003*
Rural	1.00	
Household food security at baseline		
Food insecure	2.86 [2.16, 3.79]	*<0.001*
Food Secure	1.00	
Maternal education		
Illiterate	1.88 [0.97, 3.64]	0.063
Primary	1.67 [0.86, 3.22]	0.130
Secondary and above	1.00	
Paternal education		
Illiterate	1.45 [0.84, 2.52]	0.186
Primary	1.53 [0.95, 2.46]	0.078
Secondary and above	1.00	

Parameter estimates as obtained from multivariable logistic regression model.

AOR = Adjusted Odds ratio.

Cl = Confidence interval.

Table 3 shows the results of multivariable logistic regression analyses stratified by place of residence. Variables that were persistent predictors of higher odds of chronic adolescent food insecurity across all residence places were female sex of the adolescent (P < 0.01), higher tertile of dependency ratio (P < 0.05) and household food insecurity at baseline (P < 0.001). However, household income was an independent predictor of chronic adolescent food insecurity only in the urban areas. Adolescents in the urban households with low (OR = 1.69, P = 0.008) and middle (OR = 1.70, P = 0.003) income tertiles were more likely to suffer from chronic food insecurity compared with those in high income tertile. The highest grade completed by the adolescent was negatively associated with chronic adolescent food insecurity (OR = 0.47, P = 0.002) in the urban areas, but not in the semi-urban and rural areas.

**Table 3:** Parameter estimates from multivariate logistic regressions predicting the odds of adolescents suffering from chronic food insecurity, stratified by residence, in Southwest Ethiopia from 2005 to 2007

**Predictors**	**Urban**		**Semi-urban**		**Rural**	
	**AOR [95% CI]**	**P**	**AOR [95% CI]**	**P**	**AOR [95% CI]**	**P**
Sex						
Male	1.00					
Female	1.45[1.10, 1.92]	*0.009*	1.55[1.17, 2.06]	*0.002*	1.58[1.19, 2.10]	*0.001*
Educational status of adolescents						
Primary (grade 0-8)	1.00		1.00			
Secondary and above (grade >9)	0.47[0.29, 0.75]	*0.002*	0.73[0.44, 1.18]	*0.198*	0.94[0.56, 1.58]	*0.818*
Household income (tertiles)						
Low	1.69[1.15, 2.50]	*0.008*	1.15[0.78, 1.71]	*0.477*	1.22[0.93, 2.81]	*0.090*
Middle	1.80[1.23, 2.63]	*0.003*	1.34[0.91, 1.96]	*0.140*	0.70[0.39, 1.26]	*0.236*
High	1.00		1.00		1.00	
Dependency ratio (tertiles)						
Low	1.00		1.00		1.00	
Middle	1.08[0.75, 1.56]	*0.689*	1.06[0.73, 1.54]	*0.772*	1.17[0.80, 1.72]	*0.413*
High	1.48[1.02, 2.14]	*0.039*	1.49[1.02, 2.17]	*0.038*	1.48[1.01, 2.17]	*0.047*
Sex of the household head						
Male	1.00		1.00		1.00	
Female	1.14[0.80, 1.61]	*0.466*	1.43[1.00, 2.05]	*0.053*	1.95[1.35, 2.83]	*<0.001*
Household food security at baseline						
Food insecure	1.00		1.00		1.00	
Food Secure	2.59[1.95, 3.45]	*<0.001*	2.71[2.03, 3.61]	*<0.001*	2.43[1.82, 3.24]	*<0.001*

Parameter estimates as obtained from multivariable logistic regression model.

AOR = Adjusted Odds ratio.

Cl = Confidence interval.

Table 4 presents the results of chronic adolescent food insecurity by chronic household food insecurity status and place of residence. Larger proportions of adolescents in chronically food insecure urban (27.3%) and semi-urban (27.8%) households were chronically food insecure (P < 0.001). However, there was no significant difference in chronic food insecurity of adolescents among rural households (P = 0.189).

**Table 4:** Chronic adolescent food insecurity in the total sample by household chronic food insecurity and place of residence

**Place of Residence**	**Household chronic food security status**	**Adolescent chronic food insecurity status**		**P**
		**Food secure**	**Food insecure**	
Urban	Food secure	94.8%	5.2%	*<0.001*
	Food insecure	72.7%	27.3%	
Semi Urban	Food secure	91.0%	9.0%	*<0.001*
	Food insecure	72.2%	27.8%	
Rural	Food secure	88.3%	11.7%	*0.189*
	Food insecure	84.8%	15.2%	

Chronic food insecurity refers to food insecurity in both year 1(2005/6) and Year 2(2006/7) of the survey for both households and adolescents.

P values are comparing food insecure adolescents with food secure adolescents of the same sex by income tertile for urban, semi-urban and rural residences.

Further analysis of the trend of chronic adolescent food insecurity by household income tertiles and place of residence for adolescents from chronically food insecure household showed a significant inverse association of household income with chronic adolescent food insecurity in the urban households (P = 0.024). As income decreased from high to low tertiles, there was a significant increase in the frequency of chronically food insecure adolescents showing the decline in the resilience of buffering as income decreased (Table 5). In gender stratified analysis, a higher proportion of girls within chronically food insecure households were chronically food insecure compared with boys in chronically food insecure households at all places of residences and income tertiles, showing that boys were better buffered (Table 6). Household income was negatively correlated with chronic food insecurity (r^2^ = -0.104).

**Table 5:** **Decline in buffering**^**†**^**of adolescents from chronic food insecurity within chronically food insecure households by place of residence as income Tertile declines**

**Place of residence**	**Household income (Tertiles in ETB)**	**Adolescents chronically food insecure**		**P**
		**n**	**%**	
Urban (n = 249)	High (76-1768)	99	18.2	*0.024*
	Middle (29-75)	81	35.8	
	Low (< 29)	69	30.4	
Semi-urban (n = 198)	High (80-2450)	54	25.9	*0.938*
	Middle (29-79)	67	28.4	
	Low (< 29)	77	28.6	
Rural (n = 263)	High (80-500)	9	33.3	*0.228*
	Middle (29-79)	104	12.5	
	Low (< 29)	150	16.0	

Jimma Longitudinal Family Survey of Youth; Round 1, 2005-2006; Round 2, 2006-2007 (N = 1911).

ETB: Ethiopian Birr.

^†^Buffering refers the proportion of ***food secure*** adolescents within chronically food insecure households.

P values compare food insecure adolescents with food secure adolescents by income tertile for urban, semi-urban and rural residences.

Percentages are from row totals.

**Table 6:** Trend of chronic adolescent food insecurity within chronically food insecure households by sex of adolescents, place of residence and household income

**Place of Residence**	**Household income**	**Girls chronically**	**Boys chronically**
	**Tertiles**	**Food Insecure (n = 146)**	**Food insecure (n = 121)**
Urban	High	23.1%	12.8%	
	Middle	40.0%	31.7%	
	Low	37.1%	23.5%	
Semi Urban	High	35.7%	15.4%	
	Middle	50.0%	12.8%	
	Low	31.6%	25.6%	
Rural	High	10.0%	0.0%	
	Middle	18.0%	7.4%	
	Low	17.7%	14.8%	

Jimma Longitudinal Family Survey of Youth; Round 1, 2005-2006; Round 2, 2006-2007.

Percentages are out of row totals showing only those who were chronically food insecure for boys and girls separately.

## Discussion

The findings of this study show that urban adolescents in low income households are more at risk of suffering from chronic food insecurity than semi-urban and rural adolescents, ceteris paribus. We also found that chronic food insecurity is much higher among adolescents who are members of households in the lower income tertile due to low purchasing power which is highly affected by food price fluctuation. Chronic food insecurity is a problem associated with poverty, affecting social groups with the weakest and most fragile access to food, both in terms of access to social networks and safety nets or productive assets. People’s access to food depends both on the purchasing power of their income and on their non-market mechanisms such as rights to land for subsistence farming and foraging purposes. When the income level is low, households resort to a number of coping strategies to gain access to food and protect food security levels [1,7,8,15]. The coping strategies used could be food stretching (reducing the size of meal), food rationing (reducing the number of times eaten), eating low quality or cheap foods and getting food in socially stigmatized ways such as begging [15].

High food prices further erode the coping capacities of many households across the developing world. When food prices rise, populations at risk of chronic food insecurity are urban low income households and rural landless and pastoralist households which do not have direct access to food items but use money to buy food [7,8,25]. The complex relationship between the availability of financial resources in households that access food through purchasing and their food security has been well described [12,26,27]. Families depending on monthly income often have a cyclic pattern of food restrictions, with food shortages most severe at the end of the month when household resources are depleted [12,27,28]. Chronic food insecurity can be linked to poverty in a bi-directional way that can often lead to a vicious circle. Both past and present economic disadvantage are associated with food insecurity [14], and poor households are often the most vulnerable to chronic food insecurity. Chronic food insecurity in addition, deepens poverty, not just in terms of negative nutritional effects on health and livelihoods, but through the use of irreversible coping mechanisms such as sale of productive assets which make it harder for families to escape from the poverty trap [29]. There is a need for social protection that underpins both production and consumption [30] to address the global commitments to Millennium Development Goals of decreasing the prevalence of poverty and hunger. In the study area, urban poor households need to be targeted through food security interventions as they are the primary victims of the consequences of the rise in the food prices. In Ethiopia food prices have increased considerably following the global food crisis. To respond to the consequences of rapid increase in food prices, the Ethiopian government has initiated a market stabilization program since 2007 [25]. Our results imply that the effectiveness of such intervention would be maximized if the urban poor are targeted.

In food constrained circumstances adults decrease their food intake to protect their children from food insecurity [16,31-33]. However, this might not guarantee that children’s food intakes are unaffected in such circumstances. The resilience of this buffering also becomes seriously challenged as the duration of food insecurity gets prolonged. This is especially noticeable in the low income urban households. In our study, chronic food insecurity increased significantly among urban adolescents who were members of food insecure households as income declined from high to low tertiles. Such a trend was not observed among rural and semi-urban adolescents. This finding suggests that the protection of adolescents from chronic food insecurity diminishes especially in the urban households as purchasing power gets further constrained.

As adolescents are in a phase of rapid growth and development the impact of chronic food insecurity on their well-being is considerable. In food insecure situations, as food becomes the first priority, expenditures on other goods and services could also be forgone to spare money for buying food [12]. This exposes population groups with the high nutritional requirements, including young children, adolescents, pregnant and lactating women to malnutrition [10]. Reductions in dietary quantity and quality are associated with many adverse health consequences [19,34-39], and these impacts may be magnified when food insecurity is chronic.

The study also demonstrated that a larger proportion of girls were chronically food insecure than boys in all residence places. This findings supports a male gender biased buffering of adolescent against food insecurity reported from the same cohort [16]. In developing countries boys are often favored in the allocation of resources within the household for cultural reasons [40-44].

Higher educational status of adolescents was negatively associated with the likelihood of having chronic food insecurity, only for urban adolescents. Education contributes to both income and knowledge and those adolescents who had better education may have better access to employment and hence to food. The strong relationship between food insecurity and lack of education has also been well documented by other studies [45,46]. However, in this study although paternal and maternal educations were negatively associated with chronic adolescent food insecurity, the association disappeared in multivariable analysis, suggesting that its effect is mediated through other variables including household income.

Our analysis also showed that a high dependency ratio was an independent predictor of chronic food insecurity among adolescents across all urban, semi-urban and rural residences. When the number of dependents is high compared to income earners, household level food availability is constrained due to increased consumption compared to availability of food. Coupled with an increase in the cost of food items, the purchasing power of the family will not be sufficient to obtain enough food for all members. Food assistance programs could play a protective role for children and adolescents in low income households in such circumstances [47]. A report from Southern African case studies by Devereux 2002 [48] showed that “*even tiny income transfers invested in income-generating activities, education, social networks, or the acquisition of productive assetscan make a difference*”, suggesting that social safety nets can play a significant role in reducing poverty and chronic food insecurity. Food insecurity is a complex phenomenon and varies through a continuum of successive stages as conditions become more severe. Therefore, there is a need for timely intervention before the situation further deteriorates.

Seasonal variation of food availability could affect the level of food insecurity in the study population. However, as the data were collected over three seasons, and each adolescent was interviewed about food security levels at the same season of the year, seasonality should not have a noteworthy effect on the findings. Our analyses also showed that there was no significant difference in chronic food insecurity between seasons (Table 1).

This study used longitudinal data to document predictors of chronic food insecurity in the context of rising food prices in Ethiopia and highlighted how increasing food prices erode intra-household buffering (protection mechanisms). However, although the food security items were adopted from scales used in developing countries and have high internal consistency [Cronbach’s alpha = 0.81], the fact that they are not validated locally might lead to a problem of misclassification. However, the fact that they vary with socioeconomic variables consistently in a plausible way indicates that this possibility is remote.

## Conclusions

The results showed that adolescents from urban areas who are members of low income households are at greater risk of chronic food insecurity. An increase in the proportion of chronically food insecure adolescents only in urban areas as income declines from high to low tertiles suggests that the resilience of buffering is eroded as purchasing power diminishes. Female sex, primary level education, high household dependency ratio were also independent predictors of chronic adolescent food insecurity regardless of place of residence. Food security interventions including safety net and market stabilization programs should target the urban low income households to reduce the extent of chronic food insecurity among adolescents.

## Competing interests

The authors declare that they have no competing interests.

## Authors’ contribution

The authors’ responsibilities were as follows: DL, TB, AG, CJ, LH designed and supervised the study and ensured quality of the data and made a substantial contribution to the local implementation of the study and LH, PK, CL, DL & MKH assisted in the analysis and interpretation of the data. TB, the corresponding author did the analysis & wrote the manuscript and had the responsibility to submit the manuscript for publication. All authors read and approved the final manuscript.

## Pre-publication history

The pre-publication history for this paper can be accessed here:

http://www.biomedcentral.com/1471-2458/12/604/prepub
